# Rapid and Economic Baculovirus Titer Determination Using a Novel Transgenic Sf9-QE Cell Line

**DOI:** 10.3390/insects16040426

**Published:** 2025-04-17

**Authors:** Hyuk-Jin Moon, Hyun-Jung Kim, Dong-Hyun Lee, Seo-Yeong Mun, Soo-Dong Woo

**Affiliations:** 1 Department of Agricultural Biology, College of Agriculture, Life & Environment Science, Chungbuk National University, Cheongju 28644, Republic of Korea; qlfmzks@chungbuk.ac.kr (H.-J.M.); ajee0216@naver.com (H.-J.K.); 1869oz80@gmail.com (D.-H.L.); msyoung7728@naver.com (S.-Y.M.); 2 IPBL Inc., Cheongju 28644, Republic of Korea; 3 Biomedical Research Institute, Chungbuk National University Hospital, Cheongju 28644, Republic of Korea

**Keywords:** baculovirus, virus titer, direct titration, Sf9-QE

## Abstract

A baculovirus expression system (BES) for the production of recombinant proteins requires rapid and easy virus titer determination. In this study, a novel direct titration method was developed using a novel Sf9-QE cell line to easily and economically determine virus titers in a short time. This direct titration method can determine virus titers by directly counting the initially infected cells. This method requires the rapid identification of the initial virus-infected cells. The genome of Sf9-QE cells, which fluoresce upon virus infection, was found to contain at least seven copy numbers of the enhanced green fluorescent protein (EGFP) transgene. This result suggests that Sf9-QE cells in the early stages of virus infection can be identified by the high expression of EGFP. It was also shown that for accurate virus titration using the direct titration method, Sf9-QE cells should be used within 3 d of subculturing. Additionally, counting fluorescent cells to establish virus infection should be performed within 15 to 30 h after virus infection for reliable virus titration. The direct titration method using Sf9-QE cells provides a rapid, reliable, and cost-effective alternative for determining baculovirus titers in BES research.

## Simple Summary

This study introduces a novel direct titration method for rapidly and economically determining baculovirus titers using a specially engineered Sf9-QE cell line. Sf9-QE transgenic cells were used because they express fluorescence early after viral infection, providing a prompt phenotype; this rapid expression was confirmed to be due to the integration of at least seven copies of the transgene into the cell genome. In addition, the direct titration method was validated using Sf9-QE cells in the three days after subculturing, demonstrating that efficient viral titer quantification is possible between 15 and 30 h after viral infection. Compared to conventional titration methods, similar titer values were obtained, indicating that the time required for protein production using recombinant baculoviruses can be significantly reduced. This new virus quantification method will be very useful in the production of and research into various medically and pharmaceutically useful recombinant proteins using insect viruses.

## 1. Introduction

The baculovirus expression system (BES), which utilizes entomopathogenic baculoviruses and insect cells, is a highly valuable system for the production of various recombinant proteins [1]. Compared to other host-based expression systems, the BES offers several advantages, including cost-effectiveness, biosafety for researchers, and the ability to provide post-translational modifications of recombinant proteins similar to those found in mammalian expression systems [2]. For the efficient production of target proteins in a BES, it is crucial to establish optimal viral infection conditions [3]; therefore, optimizing the multiplicity of infection (MOI)—the degree of viral infection—is essential to ensure the efficient production of target proteins by maintaining a balance between cell viability and virus production [4].

Accurate virus titration is essential for optimizing the MOI during virus infection. Various virus titration methods, including plaque assays [5], end-point dilution assays involving TCID_50_ (tissue culture infective dose 50) [6], quantitative PCR (qPCR) [7], flow cytometry [8], and enzyme-linked immunosorbent assays (ELISAs) [9], have been developed and are widely used; however, each method has its limitations, such as specific equipment requirements, technical difficulties, or accuracy issues related to experimenter proficiency. Among these methods, the end-point dilution assay involving TCID_50_ is the most commonly used. This method calculates the virus titer by performing serial dilutions of the virus and infecting insect cells, followed by determining the ratio of infected to uninfected wells [10]. This method also allows for accurate virus titer determination even at very low virus concentrations and provides statistically reliable data; however, because this method statistically determines virus titers based on the observation of the cytopathic effect (CPE), it relies on visual observation to determine infection, which introduces subjective interpretation by experimenters, leading to potential variability in the results [6].

To overcome the limitations of the described methods, a transgenic cell line, Sf9-ET, was developed based on the Sf9 insect cell line, which can express enhanced green fluorescent protein (EGFP) upon infection with a baculovirus [11]. This allowed for the relatively easy identification of virus infections under fluorescence microscopy compared to the CPE. Nevertheless, challenges remained, including the long time required for titration due to low fluorescence expression. Hence, the Sf9-QE transgenic cell line was developed to overcome this shortcoming [12]. Sf9-QE cells utilize a combination of regulatory elements, including the homologous region 3 (hr3), *39k* promoter, and *p10* promoter, for a more efficient expression of EGFP. As a result, Sf9-QE cells can complete titration earlier than Sf9-ET cells due to the rapid and strong expression of EGFP; however, the final virus titer determination still requires 6.0 to 6.3 days.

**Figure 1:**
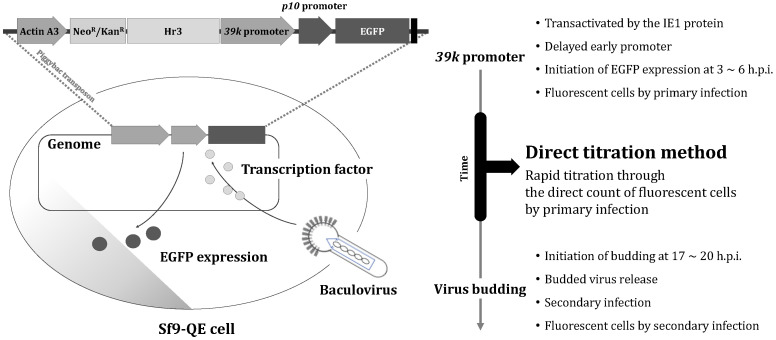
Principle and strategy of the direct titration method for baculovirus titer determination using Sf9-QE cells. Sf9-QE cells fluoresce only upon viral infection. In Sf9-QE cells, the EGFP gene is expressed by the early promoter of the virus, so fluorescence can occur early after viral infection. Secondary viral infection (cell-to-cell infection) can occur from 17 h after the primary viral infection; therefore, theoretically, it would take at least 20 h for the Sf9-QE cells to fluoresce upon secondary viral infection. Virus titers can be determined by directly counting fluorescent cells upon primary infection before fluorescence occurs upon secondary infection.

Therefore, in this study, we aimed to develop a new titration method, the direct titration method, with a dramatically reduced time required to determine titers using the Sf9-QE cell line (Figure 1). In the plaque assay, the virus is directly titrated by the number of plaques formed after serial dilution and the inoculation of the virus to be titrated [5]. Using the same principle, our hypothesis was that virus titer determination would be possible if the number of virus-infected cells could be directly counted after serial dilutions of the virus to be titrated; however, unlike the plaque assay, reliable virus titer determination was expected to be possible only if the phenotype identified with EGFP during the primary infection in Sf9-QE cells could be confirmed before the phenotype after the secondary infection of the virus. The observation of EGFP expression in the Sf9-QE cells as early as one day post-infection (d.p.i.) showed the feasibility of such virus titer determination [12]. It has been reported that the budding of baculoviruses occurs from about 17 to 20 h after primary virus infection, with the infection of other cells and the expression of the phenotype occurring after additional time [13]. The rapid and robust expression of the phenotype is required to confirm the phenotype of the primary infection of the virus prior to the expression of the phenotype of the cells after the secondary infection. In the case of Sf9-QE, the early expression of EGFP has already been demonstrated as early as one day after viral infection by the *39k* promoter, a delayed-early promoter that is activated by the immediately early 1 (IE–1) protein and operates from 3 to 6 h after infection [14,15]. Additionally, although the rapid expression of EGFP at 1 d.p.i. was confirmed, it was believed that the copy number of the transgene present in the transfected cell line would have a major influence on the quantitative expression; therefore, the determination of the copy number of the EGFP transgene in the Sf9-QE cells would further confirm the feasibility of developing a rapid titration method using Sf9-QE cells.

This study presents a new direct titration method that utilizes the distinct properties of the Sf9-QE cell line to overcome the limitations of traditional virus titration techniques through a cost-effective, rapid process while minimizing subjectivity.

## 2. Materials and Methods

### 2.1. Cells and Viruses

The *Spodoptera frugiperda* cell line Sf9 and the transgenic cell line Sf9-QE were cultured at 27 °C using an Sf–900™ II SFM medium (Gibco, Grand Island, NY, USA) and an Sf–900™ II SFM medium supplemented with G418 (Invitrogen, Carlsbad, CA, USA), respectively. The viruses used consisted of rMultibac, rAc-polh-TEVp, rAc-polh-CVA6-P1, and rAc-polh-RSV-F. rMultibac was generated using Multibac (Geneva Biotech, Geneva, Switzerland), a bacmid derived from *Autographa californica* nucleopolyhedrovirus (AcMNPV-E2, GenBank No. KM667940.1). rAc-polh-TEVP, rAc-polh-CVA6-P1, and rAc-polh-RSV-F are recombinant viruses that express the protease of the tobacco etch virus, the P1 protein of Coxsackievirus A6, and the fusion (F) protein of the respiratory syncytial virus under a *polyhedrin* promoter, respectively (Figure S1).

### 2.2. Cell Genomic DNA Extraction

Genomic DNA extraction from Sf9-QE cells was performed using an AccuPrep Genomic DNA Extraction Kit (Bioneer, Daejeon, Republic of Korea) according to the manufacturer’s instructions. Briefly, cells were collected by centrifugation at 4000 rpm for 5 min, and the supernatant was discarded. The cell pellet was washed with PBS (1 X) and centrifuged under the same conditions. After removing the supernatant, the cell pellet was resuspended in PBS (1 X). Cells were lysed by adding the provided lysis buffer, followed by the addition of absolute ethanol to the lysate. The mixture was transferred to a spin column and centrifuged at 8000 rpm for 1 min. The column membrane was washed with the provided wash buffer and subjected to a final centrifugation at 13,000 rpm for 1 min to remove the remaining ethanol. The column was then placed into a new microcentrifuge tube, and 50 µL of nuclease-free water was added. The column was centrifuged at 8000 rpm for 1 min to elute the genomic DNA.

### 2.3. Quantitative Real-Time PCR

To estimate the copy number of the transgene, qPCR was conducted using a QuanStudio1 Real-Time PCR system (Thermo Fisher Scientific, Waltham, MA, USA) with Power SYBR™ Green PCR Master Mix (Thermo Fisher Scientific, Waltham, MA, USA) according to the manufacturer’s instructions. Genomic DNA extracted from Sf9-QE cells was serially diluted 2-fold to generate five different concentrations. Each qPCR reaction was performed in a total volume of 20 µL, consisting of 1 µL of template DNA from each concentration, 10 µL of Power SYBR™ Green PCR Master Mix, 1 µL each of forward and reverse primer for the amplification of the EGFP transgene and reference genes, and nuclease-free water to adjust the final volume. Amplification was performed with an initial denaturation at 95 °C for 2 min, followed by 40 cycles of denaturation at 95 °C for 1 s and annealing at 60 °C for 30 s, and a melt-curve analysis was conducted after amplification at 95 °C for 15 s, 60 °C for 1 min, and 95 °C for 15 s. Each reaction was performed in triplicate.

### 2.4. End-Point Dilution Assay Involving TCID_50_

To perform the end-point dilution assay involving TCID_50_ based on the Reed–Muench method [6], Sf9-QE cells were distributed at a density of 2.0 × 10^4^ cells/90 µL per well in a 96-well cell culture plate. The virus was serially diluted using an Sf–900™ II SFM medium, and 10 µL of the virus at each dilution was inoculated into 8 wells of distributed cells. Then, cells were incubated under the same conditions as described above. Six days after virus inoculation, the number of infected and uninfected wells was counted for each virus dilution using a fluorescence microscope, and the virus titer was calculated using TCID_50_.

### 2.5. Direct Titration Method

Sf9-QE cells were distributed at a density of 2.0 × 10^4^ cells/90 µL per well in a 96-well cell culture plate. The virus was serially diluted using an Sf–900™ II SFM medium, and 10 µL of the virus at each dilution was inoculated into the 3 wells (for 3 repetitions) of distributed cells. Then, the cells were incubated under the same conditions as described above, and the number of cells expressing EGFP was counted using a fluorescence microscope at specific times after infection. The virus titer was calculated by multiplying the average number of cells counted and the viral dilution ratio.

### 2.6. Statistical Analysis

All statistical analyses were performed using IBM SPSS Statistics version 25 (IBM Corp., Armonk, NY, USA). Descriptive statistics were calculated for each group, including means and standard deviations. To evaluate the significance of differences between groups, a *t*-test was conducted on triplicate experimental groups. A *p*-value of less than 0.05 was considered statistically significant.

## 3. Results

### 3.1. Estimation of Transgene Copy Number in Sf9-QE

To determine the copy number of the EGFP gene integrated into the Sf9-QE cells, glyceraldehyde–3-phosphate dehydrogenase (GAPDH) and elongation factor 1-α (EF1α) genes were selected as candidate reference genes for qPCR normalization. These genes are known to play housekeeping roles in glycolysis and translation elongation, respectively, and are widely used as reference genes due to their consistent and stable expression in Sf9 cells [16]; however, their exact copy numbers have not been reported. The design and specificity evaluation of qPCR primers for GAPDH (GenBank No. KC262638.1), EF1α (GenBank No. U2013.9.1), and EGFP genes was performed using Primer-BLAST (National Center for Biotechnology Information, https://www.ncbi.nlm.nih.gov/tools/primer-blast/ (accessed on 8 January 2025)), and the sequences of primer pairs are shown in Table S1.

**Figure 2:**
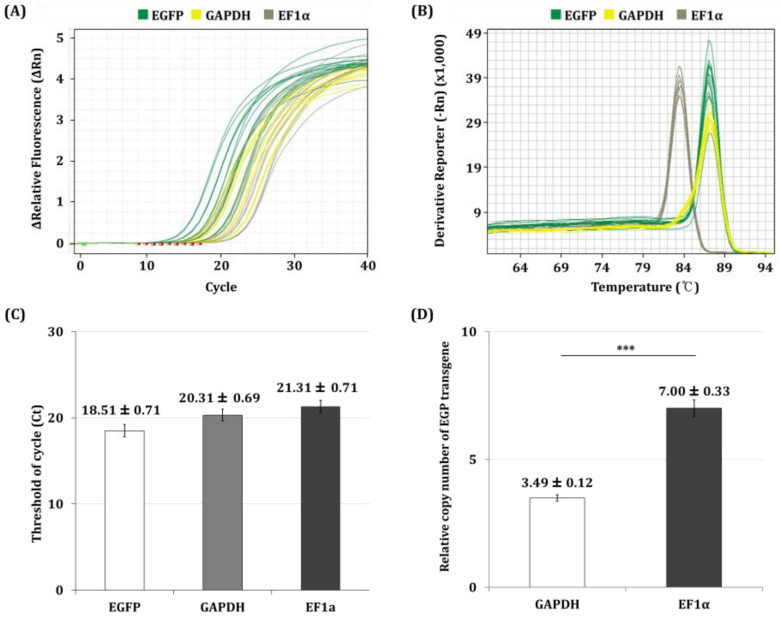
qPCR analysis of the EGFP transgene and reference genes in the Sf9-QE cell genome. To determine the relative copy number of the EGFP transgene in the Sf9-QE cell genome, qPCR was performed using GAPDH and EF1α genes as reference genes. The amplification and melt curves were analyzed to ensure specific and efficient amplification, and the copy number ratio of the EGFP transgene was determined using the ΔCt method. (**A**) Amplification plot of the EGFP transgene and the reference genes in the Sf9-QE cell genome obtained using qPCR. (**B**) Melt curves confirming the specificity of the amplified products. (**C**) Cycle threshold (Ct) values for each gene. (**D**) Calculation of the EGFP transgene copy number ratio relative to the reference genes in the Sf9-QE cell genome. Statistical significance was determined using a *t*-test (*p* < 0.05); *** *p* < 0.001.

As a result, the reliability of the qPCR was confirmed by amplification-curve and melting-curve analysis (Figure 2A,B). The cycle threshold (Ct) values of the EGFP, GAPDH, and EF1α genes were found to be 18.51 ± 0.91, 20.31 ± 0.89, and 21.31 ± 0.92, respectively (Figure 2C). The copy number ratio of the transgene was calculated using the ΔCt method, where ΔCt is defined as the difference between the Ct values of the transgene and the reference gene. The copy number ratio of the EGFP gene to each reference gene was calculated using the following equation [17]:

Copy number ratio = 2^−ΔCt^

ΔCt = Ct_transgene_ − Ct_reference gene_

Based on the calculations, the copy number ratio of the EGFP gene was determined to be 3.49 ± 0.12 compared to the GAPDH gene and 7.00 ± 0.33 compared to the EF1α gene (Figure 2D). These results indicate that the EGFP transgene is present with approximately 3.5 and approximately 7 times more copies than the respective reference genes, indicating that there are at least seven copies of the EGFP transgene in the Sf9-QE genome; therefore, the high copy number of the EGFP transgene in the Sf9-QE cells suggests that the direct- titration method in this study is likely to be successful due to a large amount of fluorescent protein production.

### 3.2. Determination of the Cell Condition for the Direct Titration Method

**Figure 3:**
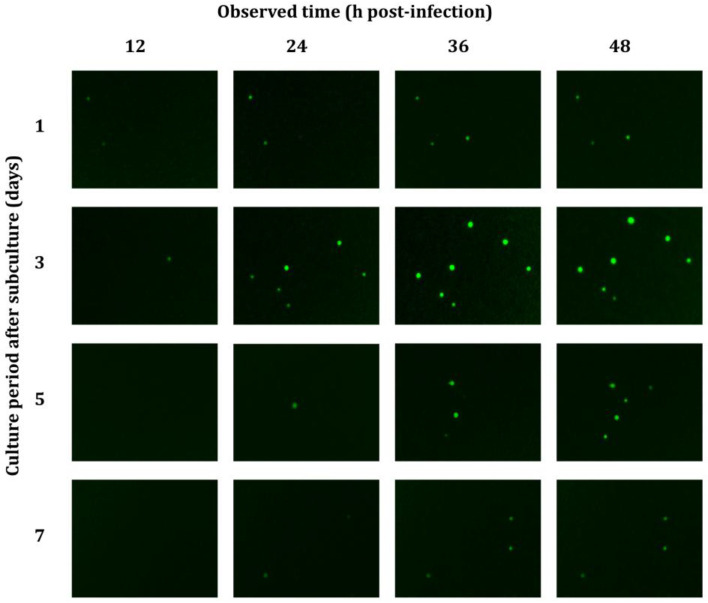
Fluorescence micrographs of Sf9-QE cells via virus infection. Sf9-QE cells were used on days one, three, five, and seven after subculturing. The virus, quantified as 2.39 × 10^8^ PFU/mL by the end-point dilution assay involving the TCID_50_, was diluted to 10–5 and inoculated into Sf9-QE cells. After virus infection, observations were made under a fluorescence microscope at 12 h intervals for up to 48 h.

Before determining the virus titer using the direct titration method, the effect of the culture condition of Sf9-QE cells on the level of EGFP expression via virus infection was evaluated. The Sf9-QE cells at one, three, five, and seven days after subculturing were infected with serially diluted viruses, and the number of fluorescent cells was observed at 12 h intervals (Figure 3). The results showed that at 24 h post-infection (h p.i.), EGFP expression was detected in the Sf9-QE cells on all days after subculturing. In particular, fluorescence was observed in cells at one and three days after subculturing, starting at 12 h p.i. The fluorescence intensity increased over time after infection and was most evident at 3 d after subculturing cells.

To determine virus titers via the direct titration method, the number of cells expressing fluorescence was counted. Virus titers were determined based on the following equation using the average number of fluorescent cells counted and the observed dilution ratio:


$$\begin{array}{l} \mathrm{Viral}\;\mathrm{titer}\;(\mathrm{number}/\mathrm{mL})=N\times 10^{D}\times 10^{\left(\mathrm{converted}\;\mathrm{µL}\;\mathrm{to}\;\mathrm{mL}\right)}\times 10^{\left(\mathrm{ratio}\;\mathrm{of}\;\mathrm{inoculation}\;\mathrm{volume}\;\mathrm{to}\;\mathrm{total}\;\mathrm{volume}\right)}\\ \;\;\;\;\;\;\;\;\;\;\;=N\times 10^{-D}\times 10^{3}\times 10^{-1}\\ \;\;\;\;\;\;\;\;\;\;\;=N\times 10^{-D+2} \end{array}$$

N—average number of fluorescent cells.
10^D^—dilution ratio of virus.

**Figure 4:**
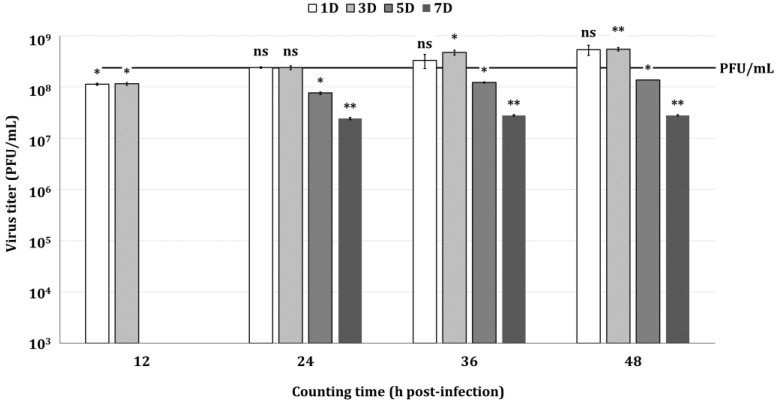
Virus quantification values by the direct titration method using the Sf9-QE cells. Sf9-QE cells were used on days one, three, five, and seven after subculturing. The virus, quantified as 2.39 × 10^8^ PFU/mL (marked as line with PFU/mL) by the end-point dilution assay involving the TCID_50_, was diluted to 10^−5^ and inoculated into the Sf9-QE cells. The number of fluorescent Sf9-QE cells was measured at 12 h intervals for up to 48 h after virus infection. Statistical significance was determined using a *t*-test (*p* < 0.05); ns (not significant, *p* ≥ 0.05), * *p* < 0.05, and ** *p* < 0.01.

The determination of virus titers by the direct titration method showed similar titers when using cells at one and three days after subculturing, but lower virus titers when using cells at five and seven days after subculturing (Figure 4). The virus titer values determined by the direct titration method were compared with the virus titers determined by the end-point dilution assay involving TCID_50_. The results showed that in cells cultured at one and three days after subculturing, the virus titers using each titration method were statistically very similar to each other. Three days after subculturing, the titers in cells were not significantly different when determined at 24 h p.i., whereas one day after subculturing, the titers were not significantly different at any time point, except at 12 h p.i.; however, at five and seven days after subculturing, significant differences in titers determined by each titration method were identified at all time points. These results indicated that the cell condition, i.e., the culture period after subculturing, is very important for the determination of virus titers via the direct titration method using Sf9-QE cells. Additionally, it was shown that there was no significant difference between the virus titer determined by the direct titration method and the PFU titer determined by the end-point dilution assay involving TCID_50_. The direct titration method for virus titration was demonstrated to be feasible using Sf9-QE cells cultured for one or three days after subculturing; however, the time range for virus titer determination by the direct titration method was reevaluated using cells three days after subculturing, which exhibited higher fluorescence intensity, allowing for easier and more accurate counting.

### 3.3. Determination of the Optimal Time for the Direct Titration Method

**Figure 5:**
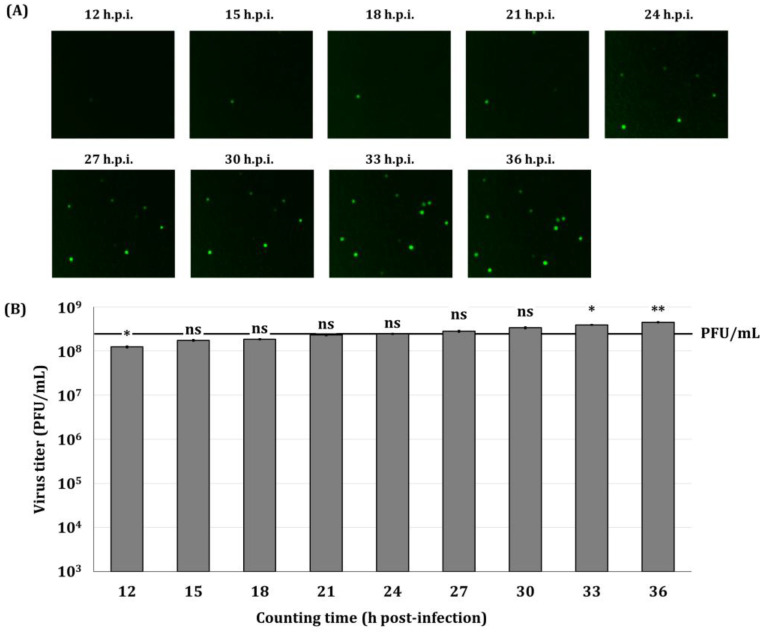
Fluorescence micrographs of Sf9-QE (**A**) with virus infection and virus quantification values (**B**) produced by the direct titration method. Sf9-QE cells at three days after subculturing were used. The virus, quantified as 2.39 × 10^8^ PFU/mL (marked as line with PFU/mL) by the end-point dilution assay involving the TCID_50_, was diluted to 10^−5^ and inoculated into the Sf9-QE cells. Fluorescence observations and cell counting were performed at 3 h intervals from 12 to 36 h after virus infection. Statistical significance was determined using a *t*-test (*p* < 0.05); ns (not significant, *p* ≥ 0.05), * *p* < 0.05, and ** *p* < 0.01.

To determine the appropriate observation time range after virus infection for accurate virus titer determination, virus titers were determined by the direct titration method using the Sf9-QE cells three days after subculturing. Virus titers were determined by observing and counting fluorescent cells at 3 h intervals from 12 to 36 h p.i. As a result, the number of fluorescent cells increased over time (Figure 5A). From 15 to 30 h p.i., the virus titers determined by the direct titration method were not significantly different from those determined by the end-point dilution assay involving TCID_50_ (Figure 5B). In particular, the virus titers determined at 24 h p.i. were the most similar. Thus, it was confirmed that the accurate determination of virus titers by the direct titration method was possible between 15 and 30 h p.i. using the Sf9-QE cells three days after subculturing.

### 3.4. Viral Titer Values Produced by the Direct Titration Method According to Virus Concentration

**Figure 6:**
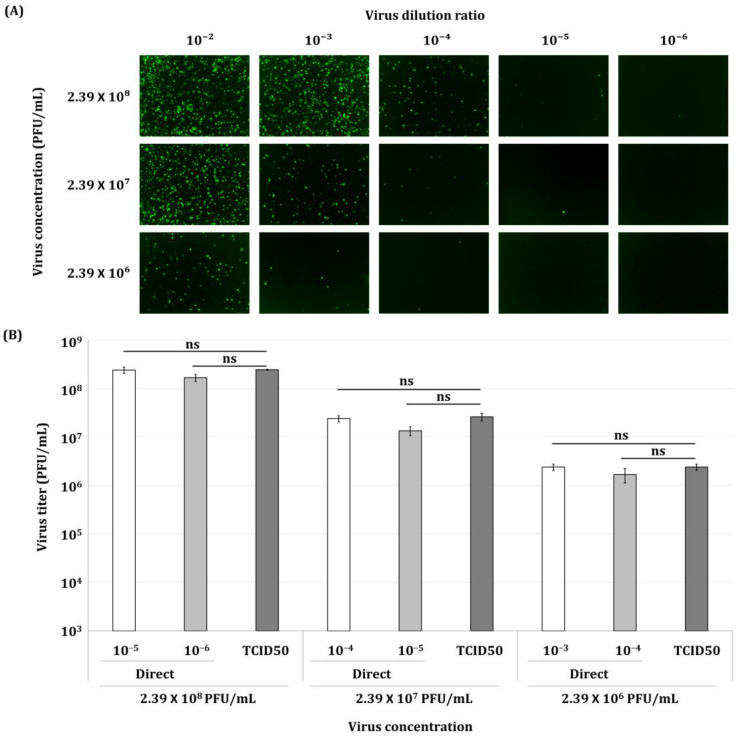
Fluorescence micrographs (**A**) of Sf9-QE cells infected with various concentrations of virus and virus quantification values (**B**) produced by the direct titration method. Sf9-QE cells at three days after subculturing were used. The virus, quantified as 2.39 × 10^8^ PFU/mL by the end-point dilution assay involving the TCID_50_, was diluted 10-fold and 100-fold. Each virus inoculum was serially diluted and then inoculated onto the Sf9-QE cells. Fluorescence observations and cell counting were performed 24 h after virus infection. Virus titers determined by the end-point dilution assay involving the TCID_50_ (TCID50) and the direct titration method (Direct) were compared. The inoculum dilution ratios for each virus used for cell counting in the direct titration method are indicated from 10^−3^ to 10^−6^. Statistical significance was determined using a *t*-test (*p* < 0.05); ns (not significant, *p* ≥ 0.05).

Whether the direct titration method for virus titer determination using the Sf9-QE cells was affected by the virus concentration to be titrated was evaluated. After infection of the Sf9-QE cells with viruses showing a 10-fold difference in virus titers and observing fluorescence expression, a higher number of fluorescent cells was observed at lower-virus-dilution concentrations (Figure 6A). Furthermore, the higher the inoculated virus concentration, the more fluorescent cells were observed, indicating that virus titrations using infected cells at higher dilutions are required to accurately count fluorescent cells. In this experiment, when 10^8^ PFU/mL of virus was inoculated, counting was easiest in inoculated wells at a 10^−5^ viral dilution, with an average of 24 cells. On the other hand, for the 10^−4^ viral dilution, counting was difficult due to the large number of fluorescent cells, with there being over 200. There was no difference in the virus quantification values between the direct titration method and the end-point dilution assay involving TCID_50_, according to the inoculated virus concentration; therefore, it was confirmed that virus titer determination using the direct titration method is possible regardless of the concentration of virus to be titrated. However, it appears necessary for the experimenter to select an appropriate virus dilution concentration that is easy to count depending on the concentration of virus to be titrated.

### 3.5. Titer Determination of Various Viruses Using the Direct Titration Method

**Figure 7:**
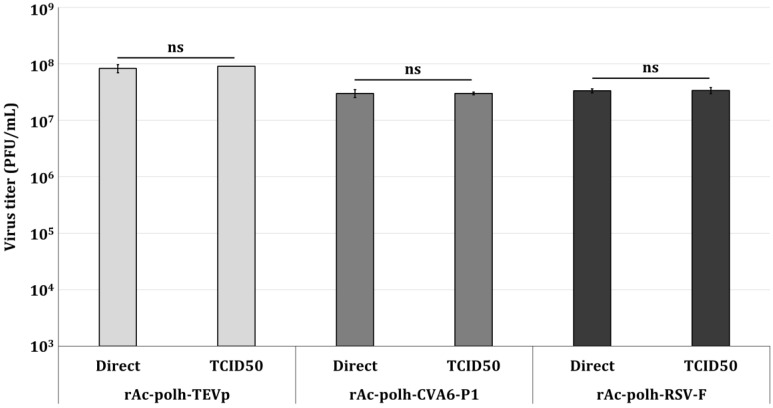
Quantitative titer values of various recombinant viruses produced by the direct titration method. The titers of recombinant viruses producing TEV-protease (rAc-polh-TEVp), CVA6-P1 (rAc-polh-CVA6-P1), and RSV-F proteins (rAc-polh-RSV-F) were determined and compared using the end-point dilution assay involving the TCID_50_ (TCID50) and the direct titration method (Direct). Statistical significance was determined using a *t*-test (*p* < 0.05); ns (not significant, *p* ≥ 0.05).

The reliability of virus titer determination by the established direct titration method using Sf9-QE cells was evaluated using a variety of recombinant viruses. The titers of all viruses tested showed no significant difference between the results obtained by the direct titration method and the end-point dilution assay involving TCID_50_ (Figure 7). These results indicate that the titers of various recombinant baculoviruses can be determined quickly and easily using the direct titration method with Sf9-QE cells.

## 4. Discussion

In this study, a novel direct titration method for virus titer determination was developed that is convenient and enables accurate titration in a short period of time—within 24 h—using unique Sf9-QE cells. Similarly to other existing virus titration methods, the direct titration method showed that virus titers can be determined by serially diluting the virus, inoculating each dilution onto 1 well of a 96-well plate, and counting infected cells before and after 24 h p.i. The reason for this convenient and rapid titration was the use of Sf9-QE cells; transgenic Sf9-QE cells can only express fluorescence upon viral infection, making it convenient to check for viral infection. In addition, the expression of the EGFP gene for fluorescence is achieved by utilizing both the *39k* promoter and strong *p10* promoter, which start to activate about 3 to 6 h p.i., allowing for rapid and high expression. Furthermore, we confirmed the presence of at least seven copies of the EGFP transgene in Sf9-QE cells, indicating that higher levels of EGFP expression may be possible. The production of a specific recombinant protein using a BES typically takes at least four–eight weeks from the generation of the recombinant virus to the identification of the target protein. Various improvements have been made to methods such as recombinant virus generation, isolation, and titration to shorten the time required to produce recombinant proteins [18,19,20]. In particular, virus titration takes at least 1 to 10 days to complete, depending on the method. Typical titration methods that can be performed in as little as one to two days include qPCR, ELISAs, and flow cytometry [7,8,9,21,22]; however, these methods have disadvantages, such as the accuracy of titration depending on the experimenter, the complexity of the experimental process, and high cost. Therefore, if it is possible to determine virus titers in a short time with a simple experimental process and low cost, it will make the BES easier and cheaper to use.

The hypothesis for this study was confirmed by the fact that accurate virus titration was possible within 15 to 30 h p.i., and that titration after 33 h p.i. was not accurate. Theoretically, given the formation time of BVs as a secondary infectious agent (17–20 h p.i.) and the early expression time of EGFP (3–6 h p.i.), fluorescence expression by secondary infection could be possible at 20–23 h p.i.; however, in our results, the accurate quantification of virus titers by direct titration was possible up to 30 h p.i. These results are believed to indicate that more time is required for the secondary infection of the virus and for verifiable levels of fluorescence to be visibly expressed. Although the accuracy of the direct titration method for determining virus titers was demonstrated in this study using repeated experiments and a variety of virus samples, it is not clear whether these results are the result of counting only fluorescent cells from primary infection, as we hypothesized.

For the direct counting of fluorescent Sf9-QE cells via primary infection with the virus, the culture status of the cells was determined to be a very important factor. Therefore, we compared the accuracy of virus titer determination by the direct titration method according to the culture period after subculturing, and showed that cells cultured within 3 days should be used. These results not only suggest that the proliferation of viruses is affected by the proliferation status of cells, but also show that a proper cell condition is required for virus titer determination by the direct titration method. According to a previous report, the proliferation of Sf9-QE cells remains in the log phase for about five to six days and then reaches the stationary phase [12]. Therefore, even Sf9-QE cells cultured for three and five days in the log phase have different effects on the proliferation of viruses, resulting in different virus titers determined by the direct titration method. Furthermore, for the direct counting of fluorescent Sf9-QE cells, counting too many cells is not only impractical, but also time-consuming. Therefore, we explored appropriate observation dilution ratios depending on the concentration of virus to be titrated and found that the concentration of virus to be titrated does not affect virus titer determination by the direct titration method. We also found that virus quantification values are reliable even at high virus dilution ratios with a minimum count of 10 or less. These results indicate that virus titer determination by the direct titration method is easier and faster; however, in our experimental experience, the selection of cells to be counted by direct titration requires some judgment on the part of the experimenter as to whether it is more appropriate to count cells at a viral dilution of 10 or less or at the dilution immediately preceding that dilution. In some cases, counting at both dilutions may be a good option.

## 5. Conclusions

In conclusion, the direct titration method for determining virus titers using Sf9-QE cells offers distinct advantages over existing virus quantification methods. It is a novel method that enables accurate virus titer determination within a short period of time, about 24 h, through a simple experimental process, with the economic advantage of not requiring expensive special equipment and using a very small amount of cells. Through this study, we demonstrated the superiority of the direct titration method for the determination of virus titers using Sf9-QE cells, and expect that this will reduce the effort and time required for BES research.

## Data Availability

The original contributions presented in this study are included in the article/Supplementary Materials; further inquiries can be directed to the corresponding author.

## Supplementary Materials

The following supporting information can be downloaded at: https://www.mdpi.com/article/10.3390/insects16040426/s1: Figure S1: Schematic representation of the recombinant viruses used in this study. Table S1: Sequences of the primer pairs used for qPCR of the transgene and reference genes in the Sf9-QE cells.
