# Supplementary material for: Rapid and Economic Baculovirus Titer Determination Using a Novel Transgenic Sf9-QE Cell Line
Source: Insects. 2025 Apr 17;16(4):426. doi: 10.3390/insects16040426 (PMC12028008; doi:10.3390/insects16040426)
Supplement: Supplementary file 1 [file insects-16-00426-s001.zip › insects-3572134-supplementary.pdf]

Supplementary Data

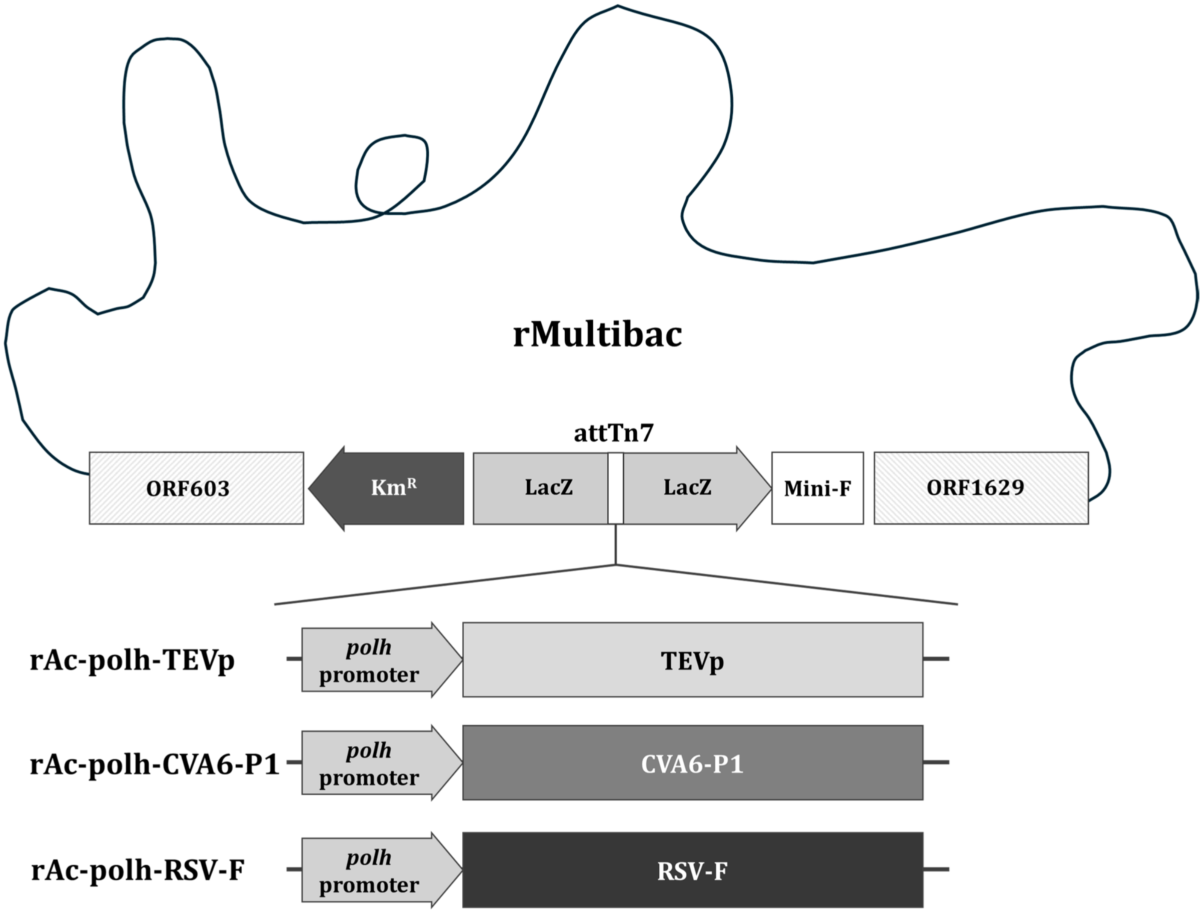

Figure S1. Schematic representation of the recombinant viruses used in this study.

Table S1. Sequences of the primer pairs used for qPCR of the transgene and reference genes in the Sf9-QE cells.

Primer	Sequences
EGFP	F: 5′- GTC CAG GAG CGC ACC ATC TT - 3′
	R: 5′- GTA CTC CAG CTT GTG CCC CA -3′
GAPDH	F: 5′- ACT GTT GAC GGA CCC TCT GG -3′
	R: 5′- CGT TAG CAA CGG GAA CAC GG -3′
EF1α	F: 5′- GTT GGA TTG CCA CAC AGC CC -3′
	R: 5′- ACA CAC AGA GGC TTG GAG GG -3′
